# Polar Localization of a Tripartite Complex of the Two-Component System DcuS/DcuR and the Transporter DctA in *Escherichia coli* Depends on the Sensor Kinase DcuS

**DOI:** 10.1371/journal.pone.0115534

**Published:** 2014-12-30

**Authors:** Patrick D. Scheu, Philipp A. Steinmetz, Felix Dempwolff, Peter L. Graumann, Gottfried Unden

**Affiliations:** 1 Institute for Microbiology and Wine Research, University of Mainz, Mainz, Germany; 2 Microbiology, Faculty for Biology, University of Freiburg, Freiburg, Germany; University of Cambridge, United Kingdom

## Abstract

The C_4_-dicarboxylate responsive sensor kinase DcuS of the DcuS/DcuR two-component system of *E. coli* is membrane-bound and reveals a polar localization. DcuS uses the C_4_-dicarboxylate transporter DctA as a co-regulator forming DctA/DcuS sensor units. Here it is shown by fluorescence microscopy with fusion proteins that DcuS has a dynamic and preferential polar localization, even at very low expression levels. Single assemblies of DcuS had high mobility in fast time lapse acquisitions, and fast recovery in FRAP experiments, excluding polar accumulation due to aggregation. DctA and DcuR fused to derivatives of the YFP protein are dispersed in the membrane or in the cytosol, respectively, when expressed without DcuS, but co-localize with DcuS when co-expressed at appropriate levels. Thus, DcuS is required for location of DctA and DcuR at the poles and formation of tripartite DctA/DcuS/DcuR sensor/regulator complexes. Vice versa, DctA, DcuR and the alternative succinate transporter DauA were not essential for polar localization of DcuS, suggesting that the polar trapping occurs by DcuS. Cardiolipin, the high curvature at the cell poles, and the cytoskeletal protein MreB were not required for polar localization. In contrast, polar localization of DcuS required the presence of the cytoplasmic PAS_C_ and the kinase domains of DcuS.

## Introduction

Bacteria reveal a complex spatial organization of some proteins within membranes or the cytoplasm, which can be represented by an accumulation of proteins at distinct sites, or in the co-localization of proteins from metabolic pathways or other cellular functions. The localization results in a spatial compartmentalization of proteins for functional reasons in bacteria which lack compartments like organelles. Co-localization and supra-molecular organisation of enzymes of respiratory chains, transport metaboloms, glycolysis and other metabolic pathways has been shown or suggested to increase, control or direct metabolic fluxes [1], [2], [3], [4], [5]. Similarly, supra-molecular organisation of sensors, co-sensors and components of signal transducing pathways in bacterial sensor complexes has been suggested to increase the sensitivity and specificity of sensors systems [6], [7], [8], [9].

Polar localization has been studied in detail for the methyl-accepting chemotaxis proteins (MCPs) within *Escherichia coli* cells [6]. MCP proteins are predominantly observed at the cell poles, where they form clusters of the MCP and other chemotaxis proteins. Cluster formation is believed to support stimulus integration and sensitivity of the sensors [10], [11]. In the same way sensor kinases involved in the regulation of cell division and development have been shown to exhibit an uneven distribution within the cell membrane which is mostly polar or at the cell septum. The subcellular localization of the corresponding histidine kinases is related to their site of function and their role in controlling processes that are located in specific cell regions [12]. For example, in *Caulobacter crescentus* the antagonistic PleC and DivJ kinases are localized at opposite cell poles and coordinate cell-cycle progression with polar differentiation [13]. The WalK (YycG) sensor histidine kinase from *Bacillus subtilis* is localized to the division septum in growing cells, thereby controlling the synthesis of proteins involved in cell wall remodelling and cell separation [14], [15].

Many sensor histidine kinases controlling metabolic processes show no obvious distinct polar distribution over the cell membrane. Thus membrane-bound histidine kinases like the citrate sensing CitS from *Bacillus subtilis*, the quorum sensing kinase LuxQ from *Vibrio harveyi*, PhoQ from the PhoQP two-component system regulating virulence in *Salmonella enterica*, and the osmosensor kinase KdpD of *E. coli* display either a heterogeneous localization at unspecified positions or a homogeneous distribution over the cell membrane [16], [17], [18], [19].

On the other hand, the (membraneous) carboxylate sensor kinases DcuS and CitA from *E. coli* accumulate at the cell poles [20]. The DcuS/DcuR two-component system induces genes required for degradation of C_4_-dicarboxylates from the medium under aerobic and anaerobic conditions [21], [22]. The major targets of transcriptional regulation by DcuS/DcuR are the genes encoding the aerobic and anaerobic C_4_-dicarboxylate transporters DctA (*dctA*) and DcuB (*dcuB*) and fumarate reductase (*frdABCD*). In addition, DcuS has been shown to interact physically and functionally with the transporter DctA that functions as a co-regulator [8]. This raises the question whether all of these components show polar localization in a DctA/DcuS complex, which might also bind the cognate response regulator DcuR. Recently, further histidine kinase sensors, i.e. TorS and EvgS, were reported to form clusters in *E. coli* predominantly at the cell poles [23].

Various mechanisms have been discussed to cause trapping at or guiding proteins to the poles [24]. Polar localization of membranous proteins can be affected by phospholipids, e.g. by trapping the proteins in cardiolipin patches which are negatively charged [25]. Cardiolipin rich patches are found at the poles and in regions of septum formation [26], and by their intrinsic curvature, these regions provide space for the arrangement of the charged head groups of cardiolipin. Localization of membrane proteins with a positive charge might be favoured in the cardiolipin rich patches around the cell poles [27]. Alternatively, structuring proteins of the cytoskeleton like MreB, or proteins directing the movement of proteins and other cell components during cell division and separation, might guide proteins to the cell poles [28], [29], [30], [31].

Studies on the establishment of such sensor complexes and of their polar localization will be a first step towards understanding the role and prerequisites for the polar localization. Therefore, the localization and co-localization of the components of the DctA - DcuS/DcuR sensor system was analysed in this study, since so far only the localization of DcuS was determined [20]. First, many cells showed polar cluster formation of DcuS when DcuS was produced at very low levels from a single chromosomal copy of *dcuS-mvenus*, driven by the native promoter, or from uninduced plasmid-borne *dcuS-yfp*. Secondly, the analysis of the dynamics of the DcuS clusters showed that the polar accumulation is observed at very low levels of the sensor and not caused by aggregation. Since DctA and DcuR interact functionally and physically with DcuS, polar localization is expected for DcuR and DctA in the sensor complex as well. The mode of polar localization shows some interesting new features. Since DctA functions in the free state as a transporter for the uptake of C_4_-dicarboxylates [8], [32], [33], only the portion of DctA participating in the DctA/DcuS sensor complex is expected to co-localize with DcuS. Therefore, polar localization of the additional constituents of the complexes (DctA and DcuR) was tested, and whether they localize independently from each other, or whether a particular protein within the complex is responsible for polar localization. Finally, the cellular and intrinsic factors controlling the localization of DcuS were analysed, since DcuS turned out to be the component of the complex responsible for polar localization.

## Materials and Methods

### 2.1 Bacteria and molecular genetic methods

The *Escherichia coli* K-12 strains and plasmids used in this study are listed in Table 1. Molecular genetic methods were performed according to standard procedures [34]. Plasmids were isolated using a QIAprep spin miniprep kit, and PCR products were purified with a QIAquick PCR purification kit (Qiagen, Hilden, Germany). *E. coli* strains were transformed by electroporation [35]. The *dcuS-cfp*, *dcuS-yfp*, *citA-yfp*, and *dctA-yfp* fusions, and *yfp* in pBAD30 were constructed as described previously [8], [20], [36]. The *dcuS*(-PASc)*-yfp*, and *dcuS*(-TM2)*-yfp* fusions were constructed similar as described for *dcuS-yfp* [20] with slight alterations. The truncated gene variants *dcuS*(-PASc) and *dcuS*(-TM2) were amplified by PCR from plasmid pMW151 with oligonucleotide primers dcuSpetCfor and PAScSacIrev (5′-CGCTGCATGAGCTCACGTAC-3′) or dcuSpetCfor and TM2SacIrev (5′-CGAAAAGGAGCTCTTTCAGTACC-3′), respectively. Either PCR fragment was cloned into pMW391 via NdeI and SacI, resulting in pMW1042 encoding *dcuS*(-PASc)*-yfp* and pMW1043 encoding *dcuS*(-TM2)*-yfp*, respectively. The fusion genes were finally cloned from pMW1042 or pMW1043 via XbaI into pBAD30, resulting in pMW1060 and pMW1061. The constructs encode His_6_-DcuS_PASc_(1-328)-(EL)-YFP(4-240) referred to as DcuS(-PASc)-YFP, and His_6_-DcuS_TM2_(1-206)-(EL)-YFP(4-240) referred to as DcuS(-TM2)-YFP. In the case of the *dcuS(ΔPASc)-yfp* construct PASc was deleted via added XhoI sites from plasmid pMW407. The resulting plasmid pMW1302 codes for His_6_-DcuS(1-218/326-543)-(EL)-YFP. The *dcuR* gene for the *yfp-dcuR* construct was cloned from pMW180 via HindIII and an additional N-terminal EcoRI restriction site into plasmid pMW392, resulting in plasmid pMW1742. Furthermore a SpeI restriction site was introduced between the EcoRI site and *dcuR* for linker ligation. The 5′ phosphorylated oligonucleotides linker_for (5′-AATTCAACAATAACAATCTCGGAATCGACACTACGGAGAACCTGTATTTCCAGGGTATGTCGTCTGGCCTAGTCCCACGTGGCAGCGCTGGTAGTAGGGGAGCAA-3′) and linker_rev (5′-CTAGTTGCTCCCCTACTACCAGCGCTGCCACGTGGGACTAGGCCAGACGACATACCCTGGAAATACAGGTTCTCC GTAGTGTCGATTCCGAGATTGTTATTGTTG-3′) were hybridized and ligated into pMW1742 via EcoRI and SpeI restriction sites. The *yfp-dcuR* and *yfp-linker-dcuR* fragments were subsequently integrated into pMW643 via an extra C-terminal XbaI restriction site. Resulting plasmids pMW1741 and pMW1953 encode for the His_6_-YFP(1-238)-(EF)-DcuR(1-239) and His_6_-mYFP(1-238)-(linker)-DcuR(1-239) constructs respectively. All additional restriction sites as well as the monomeric YFP variant A206K were introduced by site-directed mutagenesis. The *dcuS-mvenus* fusion was constructed and inserted in the chromosome of *E. coli* as described in the Supplemental Information; the gene fusion finally encodes DcuS(1-543)-(GH)-mVenus(1-240). The sequences of all resulting constructs were verified by DNA sequencing.

**Table 1:** Strains of *Escherichia coli* and plasmids used in this study.

Strain or plasmid	Genotype	Reference or source
*E. coli* K-12 strains		
EK1	BW25113, but Δ*dauA*	[41]
MC4100	F^−^ *araD139* Δ(*argF-lac*)*U169*, *rpsL150*, *relA1 flbB530 deoC1 ptsF25 rbsR* Δ*lacZ*	[64]
MDO800	AN387, but *dctA::*Spc*^r^*	[65]
JM109λ*pir*	JM109 *pir^+^*	[66] Keio Collection
JW1241	BW25113, but *cls::*Kan*^r^*	[66] Keio Collection
IMW237	MC4100, but λ[Φ(*dcuB-lacZ*)hyb *bla* ^+^]	[22]
IMW238	MC4100, but λ[Φ(*dcuB-lacZ*)hyb *bla* ^+^] *dcuR*::Kan^r^	[22]
IMW260	MC4100, but λ[Φ(*dcuB-lacZ*)hyb *bla* ^+^] *dcuS::*Cam*^r^*	[22]
IMW262	MC4100, but *dcuS::*Cam*^r^*	[22]
IMW279	MC4100, but *citA::*Kan*^r^*	[67]
IMW570	MC4100, but *dcuS::dcuS-bs2*	This study
IMW612	IMW237, but *dcuS::dcuS-mvenus*	This study
BKT12	W3110, but *ΔclsA*, *ΔclsB*, *ΔclsC*::Kan^R^	[44]
W3110	F^−^ *λ^−^ IN(rrnD-rrnE)1 rph-1*	*E. coli,* Genetic Stock, Center, Yale University
**Plasmids**		
pBAD18-Kan	Expression vector; pBR322 ori, pBAD promoter (Kan^r^)	[68]
pBAD30	Expression vector; pACYC ori, pBAD promoter (Ap^r^)	[68]
pDS132	Suicide vector; *R6K ori*, *sacB*, *mobRP4* (Cam^r^)	[69]
pDS132::*dcuS-mvenus*	Suicide vector for chromosomal insertion of *dcuS-mvenus* (Cam^r^)	This study
pET28a	Expression vector; pBR322 ori, T7 promoter, His tag (Kan^r^)	Novagen
pGlow-Bs2-stop	Bs2 expression vector (Ap^r^)	[70] Evocatal evoglow
pMW151	DcuS expression plasmid; pET28a derivative (Kan^r^)	[71]
pMW180	DcuR expression plasmid; pET28a derivative (Kan^r^)	[71]
pMW181	DcuS expression plasmid; *dcuS* expressed from its native promoter; pET28a derivative (Kan^r^)	[72]
pMW384	DcuS-YFP expression plasmid; pMW391 derivative (Kan^r^)	[20]
pMW391	*‘yfp*, protein fusion plasmid; pET28a derivative (Kan^r^)	[20]
pMW392	*Yfp‘*, protein fusion plasmid; pET28a derivative (Kan^r^)	This study
pMW407	DcuS-YFP expression plasmid; pBAD30 derivative (Ap^r^)	[20]
pMW408	DcuS-CFP expression plasmid; pBAD18-Kan derivative (Kan^r^)	[36]
pMW442	CitA-YFP expression plasmid; pBAD30 derivative (Ap^r^)	[20]
pMW526	DctA-YFP expression plasmid; pBAD30 derivative (Ap^r^)	[8]
pMW643	pBAD30 with additional Tet^r^	[36]
pMW765	YFP expression plasmid; pBAD30 derivative (Ap^r^)	[36]
pMW842	*dcuS-bs2-stop-3′dcuS*; pUC18 derivative (Ap^r^)	This study
pMW843	*’dcuS-bs2-stop*-*3′dcuS*; pDS132 derivative (Cam^r^)	This study
pMW875	DcuS-Bs2 expression plasmid; pMW643 derivative (Ap^r^ Tet^r^)	This study
pMW1042	DcuS(-PASc)-YFP expression plasmid; pET28a derivative (Kan^r^)	This study
pMW1043	DcuS(-TM2)-YFP expression plasmid; pET28a derivative (Kan^r^)	This study
pMW1060	DcuS(-PASc)-YFP expression plasmid; pBAD30 derivative (Ap^r^)	This study
pMW1061	DcuS(-TM2)-YFP expression plasmid; pBAD30 derivative (Ap^r^)	This study
pMW1302	DcuS(ΔPASc)-YFP expression plasmid; pBAD30 derivative (Ap^r^)	This study
pMW1390	DcuS expression plasmid; pBAD18-Kan derivative (Kan^r^)	[8]
pMW1739	DcuR-YFP expression plasmid; pMW643 derivative (Ap^r^ Tet^r^)	This study
pMW1740	DcuR expression plasmid; pMW643 derivative (Ap^r^ Tet^r^)	This study
pMW1741	YFP-DcuR expression plasmid; pMW643 derivative (Ap^r^ Tet^r^)	This study
pMW1742	YFP-DcuR expression plasmid; pET28a derivative (Kan^r^)	This study
pMW1891	DcuS-mYFP(A206K) expression plasmid; pMW407 derivative (Ap^r^)	This study
pMW1953	mYFP(A206K)-linker-DcuR expression plasmid; pMW643 derivative (Ap^r^ Tet^r^)	This study
pSG1164::*dcuS-mvenus*	Vector harbouring *mvenus* (Ap^r^ Cam^r^)	[73]

### 2.2 *E. coli* expressing fluorescent proteins

Expression plasmids with fluorescent protein fusions were transformed into *E. coli* strains. Bacteria were grown aerobically in LB medium at 30°C. Overnight cultures were diluted 1/50 or 1/100 in fresh medium and incubated for 2–3 h, corresponding to the mid-exponential phase of growth, and induced from the beginning of incubation with 133 µM L-arabinose. For repressive conditions glucose (100 µM) was included in the medium. Main cultures were grown to mid-late exponential growth phase and subsequently induced with 133 µM L-arabinose for 1–1.5 h. Alternatively, cells were grown in enriched mineral (eM9) medium supplemented with acid-hydrolyzed casamino acids (0.1%), tryptophan (0.005%), and glycerol (50 mM) as carbon source, with and without sodium fumarate as effector. Ampicillin, kanamycin or tetracycline were added at a concentration of 100 µg/ml, 50 µg/ml or 15 µg/ml respectively. When two plasmids were coexpressed in one cell, antibiotic concentrations were halved.

### 2.3 Microscopy

For fluorescence microscopy, 5 µl of a culture grown in LB medium or in eM9 medium and washed and resuspended in 1% phosphate-buffered saline (PBS) buffer pH 7.5, were spotted on a microscope slide that was freshly coated with a thin layer of 1% agarose and covered with a coverslip. For elongation of *E. coli* cells, the cell division inhibitor cephalexin was added to exponentially growing cultures at 10 to 30 µg/ml, and bacteria were further incubated for 1–3 h. Spheroplasts of *E. coli* strain IMW262 expressing *dcuS-yfp* were obtained by treatment with Lysozyme-EDTA as described previously [37]. Where indicated, the A22 MreB-depolymerizing drug was added to exponentially growing cultures at 10 µg/ml for 2 h. Prior to FRAP experiments, the transcription inhibitor rifampicin was added to exponentially growing cultures at 50 µg/ml for 1 h. Subsequently, cells were prepared for microscopy as described above.

### 2.4 Fluorescence microscopy imaging

Fluorescence microscopy was performed using a Zeiss AX10 microscope equipped with a CoolSNAP HQ Camera (Photometrics), or a Keyence Biozero BZ-8000 microscope. Fluorescence signals were monitored using an appropriate filter cube, and images were acquired with MetaMorph 6.1 software, and processed with ImageJ software (Image Processing and Analysis). For FRAP (fluorescence recovery after photobleaching) experiments, a Zeiss Axio Observer Z1 (inverted microscope) equipped with a Cascade II 512 camera (Photometrics) and an external laser source was used. The specimen was bleached with a focussed 405 nm laser beam, and the fluorescence recovery of YFP was monitored by excitation at 488 nm. Due to low expression and signal level, fluorescence microscopy of chromosomally encoded *dcuS-mvenus* was performed using a confocal Leica TCS SP8 microscope with a 100x lens (NA 1.4) and the light source of a pulsed white-light laser.

## Results

Earlier studies have shown the (MCP-independent) polar accumulation of DcuS-YFP in *E. coli* [20], when it was expressed from a low copy plasmid. Here, the cellular localization of DcuS was investigated when it is present at extremely low levels: a) when expressed from its native chromosomal site, and b) when visualized as plasmid-born system under promoter-repressive conditions. In addition, the localization of the other components of the sensory system, that is the cognate response regulator DcuR and the regulatory transporter DctA were tested for their cellular localization. The studies with low levels of DcuS together with examining cluster dynamics of DcuS by FRAP was used to demonstrate that the polar localization is not caused by overproduction of the protein. For the same reason, all fusion proteins used in the study were tested for their functionality in complementation of expression or growth assays, respectively [20], [36]. Generally, different types of fusion proteins like DcuS-YFP, YFP-DcuS or DcuS-mVenus were active in complementation suggesting that the fusion proteins (rather than cleavage products) were responsible for the activity in complementation.

### Polar localization of DcuS at very low expression levels

It was shown earlier [20] that the polar accumulation of DcuS-YFP is found when the protein is present at low levels. For studies on the behavior of DcuS at wild-type levels of the protein, DcuS-mVenus (IMW612) was expressed from a chromosomally inserted copy of *dcuS-mvenus* using the native *dcuS* promoter. DcuS-mVenus produced in this way was functional when DcuR was available and complemented a chromosomal *dcuS* null mutant (S1 Fig.). Analysis by high sensitivity fluorescence microscopy revealed that DcuS-mVenus forms dynamic clusters at or nearby old and new cell poles (S2 Fig.), in addition to randomly positioned foci along the lateral cell membrane.

**Figure 1:**
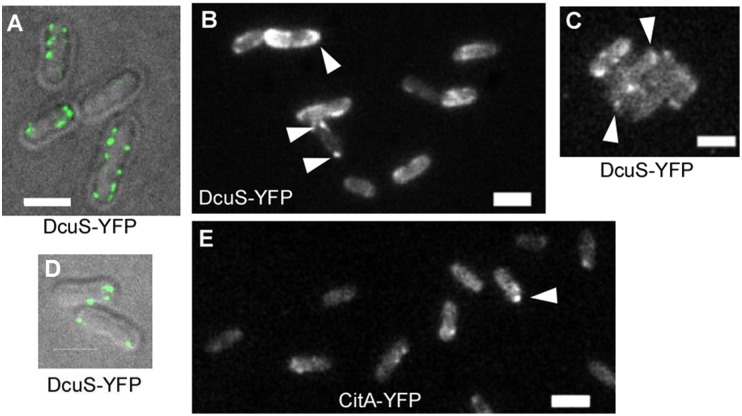
Localization of chromosomally expressed or low-level-induced DcuS-YFP in *E. coli*. Panels A and D confocal microscopy (overlays of bright field and fluorescence), panels B, C and E epifluorescence (A–C, and E exponentially growing cells). A–B) DcuS-YFP expressed from the chromosome (strain IMW612), C) DcuS-YFP expressed from a plasmid (W3110pMW407), D) stationary phase cells expressing DcuS-YFP at low level (W3110pMW407), E) cells expressing CitA-YFP (W3110pMW422). White triangles indicate polar localized protein clusters. Scale bars, 2 µm.

Similar levels of very low expression were obtained for a DcuS-YFP fusion protein when *dcuS-YFP* was expressed from an arabinose inducible promoter on the low copy vector pBAD30 without induction, and under glucose-repressive conditions. Clear signals of DcuS-YFP were detected by fluorescence microscopy (Fig. 1 A–D). The molecules were accumulated at the cell poles in about 25% of the cells (250 cells analysed), but the accumulation was transient without permanent cluster formation, and the DcuS-YFP molecules were mobile, i.e. they changed their positioning at the poles and at the lateral cell membrane between 200 ms intervals (S1 Movie). Because only few molecules will be produced under these conditions, these analysis rule out an overproduction artefact.

Under mild induction with 133 µM arabinose, the levels of DcuS-YFP increased to levels detectable by fluorescence microscopy, and more than 50% of all cells showed polar localization (see below). For convenience and easy detection of DcuS-derived fluorescence, further experiments were performed either with *dcuS-yfp* expressed from the low-copy vector pBAD30 after weak induction, or, as indicated, without induction to obtain very low levels of DcuS-YFP. Polar cluster formation of DcuS-YFP was observed during all growth phases, e.g. in early-exponential (S3 Fig.), mid-exponential, and stationary phase. The closely related sensor histidine kinase CitA has a similar preferentially polar localization pattern as DcuS-YFP (Fig. 1 E, S2 Movie).

### FRAP (Fluorescence Recovery After Photobleaching) of DcuS-YFP

**Figure 2:**
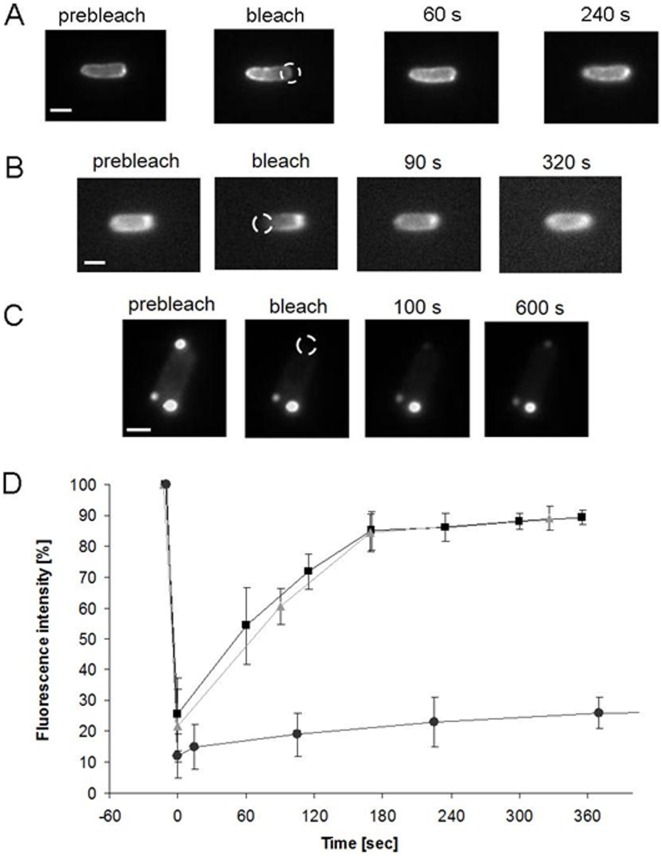
FRAP experiments of *E. coli* cells expressing (A) DcuS-YFP, (B) CitA-YFP, both showing fluorescence recovery at the cell pole, and (C) aggregated, non-functional DcuR-YFP showing no fluorescence recovery. DcuS-YFP (pMW407) was expressed in strain IMW262, CitA-YFP (pMW442) in strain IMW279, and DcuR-YFP (pMW1082) in strain IMW238, all in the presence of 133 µM arabinose. (D) The diagram depicts the relative fluorescence intensity of the fluorescent area at the cell pole before and after bleaching over time, normalized against gradual bleaching of the images, each from four independent experiments (standard deviations shown); square, DcuS-YFP; triangle, CitA-YFP; circle, aggregated DcuR-YFP. The mean half-time recovery of DcuS-YFP is 62 s, that of CitA-YFP is 80 s. Four independent experiments each were performed. The pictures illustrate representative examples of the microscopic acquisitions, with the dashed circle indicating the bleached area. Scale bars, 1 µm.

The sensor histidine kinase DcuS is present as dimer and higher oligomer in the membrane [36] with apparently high dynamics. We studied the dynamics of the polar DcuS clusters in single cells by FRAP (Fluorescence Recovery After Photobleaching). For FRAP experiments, the *E. coli* strain producing DcuS-YFP with polar accumulation was grown to the exponential growth phase with L-arabinose induction. The bacteria were treated with the transcriptional inhibitor rifampicin before starting the FRAP experiments. After bleaching the fluorescence of DcuS-YFP at one pole or in one half of the bacterial cell, respectively, the DcuS-YFP fluorescence was recovered, with similar polar accumulation as before bleaching, with a half-time recovery of 62 s (Fig. 2A, D). The recovery time is comparable to that reported for the membrane-bound chemotaxis receptors [38]. The sensor kinase CitA has a similar dynamic pattern as DcuS-YFP, and showed the same fluorescence recovery of the YFP signal as DcuS-YFP when treated in the same way (Fig. 2 B, D) with a half-time recovery of 84 sec. In a control experiment, this is contrasted by very poor and slow recovery of DcuR-YFP (Fig. 2 C, D), which is not functional and forms cytoplasmic aggregates. Thus, the clusters of DcuS and CitA reveal a dynamic cluster formation after bleaching of the existing clusters, indicating a rapid and permanent cluster turnover, even when the sensors were produced at slightly higher levels than under physiological conditions, with significant polar clustering. High turnover is in agreement with the movement of DcuS-YFP assemblies seen by time lapse microscopy.

### Polar localization of regulators for C_4_-dicarboxylate metabolism, DcuS, DcuR, and DctA

**Figure 3:**
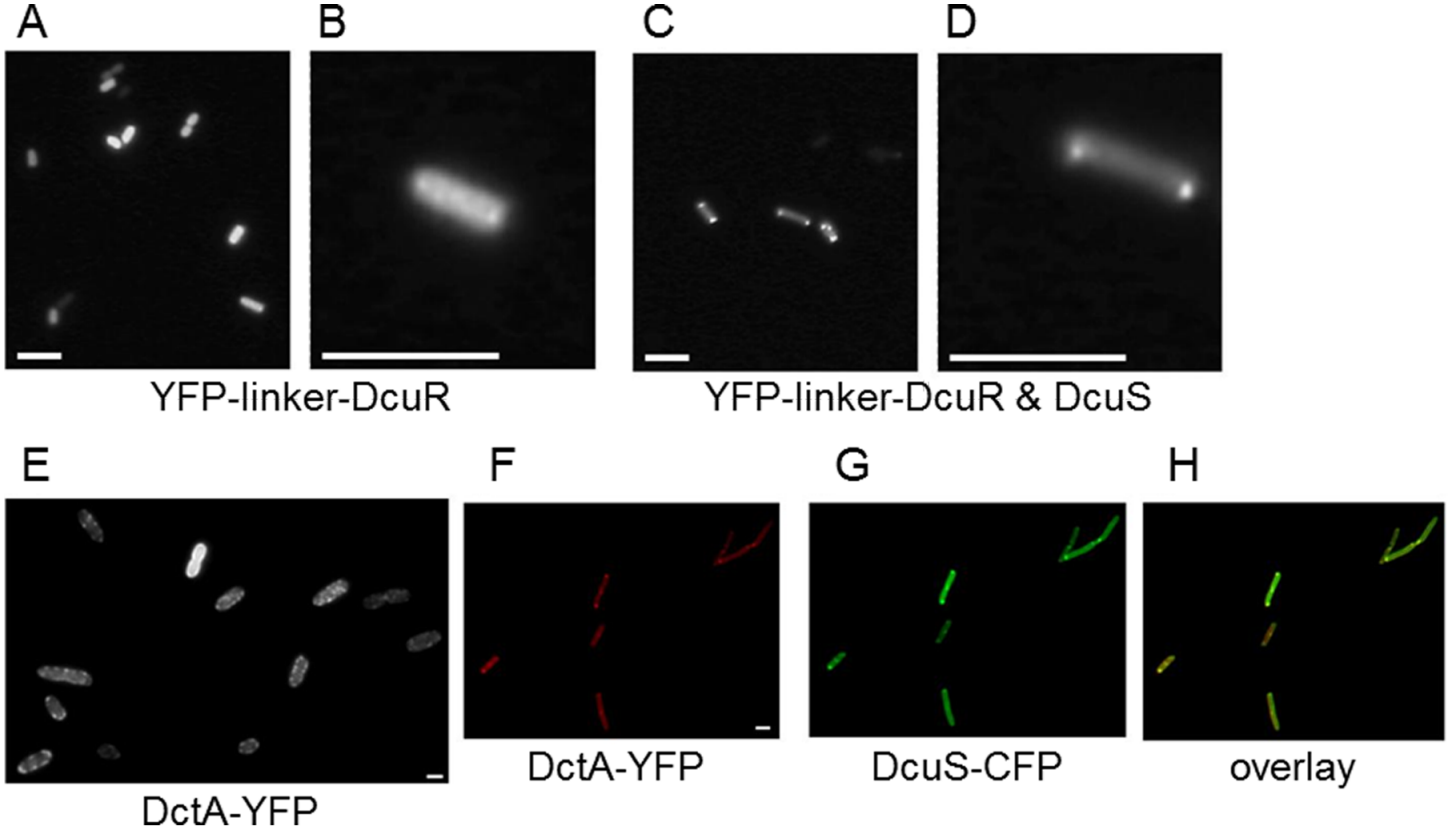
Localization of the cognate response regulator DcuR fused to YFP(A–B) and DctA-YFP(E) and their co-localization with DcuS (C–D, F–G). mYFP(A206K)-linker-DcuR fluorescence (strain IMW238/pMW1953) was visualized: (A) overview, (B) closeup; mYFP(A206K)-linker-DcuR and DcuS(pMW1390) were coexpressed: (C) overview, (D) closeup; scale bars 5 µm. (E) DctA-YFP fluorescence (strain IMW262/pMW526) was visualized; scale bar, 1 µm. For co-localization of DctA-YFP and DcuS-CFP, DctA-YFP (pMW526) and DcuS-CFP (pMW408) were coexpressed in IMW262 and fluorescence of (F) YFP (depicted in red) and (G) CFP (depicted in green) were detected separately and (H) merged (overlay image). 50 to 100 cells were inspected, with 60 to 90% showing the respective localization, scale bar, 1 µm.

The sensor DcuS shows protein interaction with the response regulator DcuR, the transporter DctA [8], [36], and with the closely related citrate sensor kinase CitA [39]. The interactions of DcuS with DcuR and DctA are relevant for the function of DcuS in sensing and signal transfer. Therefore, the cellular localization of the proteins functionally related to DcuS, in particular DctA and DcuR, was determined. The fusion of YFP to DcuR was only functional in complementation assays when YFP was separated from DcuR by a linker (YFP-linker-DcuR) whereas direct fusion (YFP-DcuR or DcuR-YFP) resulted in complementation inactive forms in a *dcuS* positive strain (S4 Fig., S5 Fig.). YFP-linker-DcuR showed a random localization over the complete cellular area (Fig. 3 A, B). A distribution of this type is characteristic for cytoplasmic localization. The localization was found *in E. coli* cells grown in the absence or presence of fumarate (not shown). When YFP-linker-DcuR was expressed in a strain where DcuS was coexpressed to the same extent (Fig. 3 C, D), the YFP-linker-DcuR fusion protein showed clear polar clusters in addition to some protein (or fluorescence) present in the cytoplasm, both when the bacteria were grown with or without fumarate. The experiments suggest that DcuR forms a complex with DcuS which might be of sufficient stability to accumulate DcuR at the site of DcuS. The accumulation becomes visible in cells expressing YFP-linker-DcuR (which is above wild-type levels, but sufficient to be visualized) only after co-expression of DcuS.

The aerobic C_4_-dicarboxylate transporter DctA functions as a co-regulator of DcuS. DctA directly interacts with DcuS and forms a functional transporter/sensor unit [8], [9], [40]. DctA-YFP displayed a homogeneous distribution in the membrane over the complete cell surface of *E. coli* in wild-type and *dcuS* mutant cells (Fig. 3 E), independent of the absence or presence of fumarate. The fluorescence had a spotty appearance, but without polar accumulation. When, however, DctA-YFP was co-expressed in a strain producing DcuS-CFP at increased levels, the distribution of DctA over the cell membrane was altered to a polar co-localization (Fig. 3 F). Normally, the cellular contents of DcuS as a sensor protein are significantly lower than those for catabolic enzymes or transporters like DctA, and it can be assumed that only after over-production of DcuS, a sufficient amount of the sensor protein is available for binding (most) of DctA, which appears to be the prerequisite for polar accumulation of DctA that accumulates only at the pole when associated with DcuS. Overlays of the fluorescence of DctA-YFP (Fig. 3 F) and DcuS-CFP (Fig. 3 G) showed clear co-localization under these conditions (Fig. 3 H). In control experiments for co-localization, free YFP was not co-localized with DcuS-CFP (S6 Fig.).

**Figure 4:**
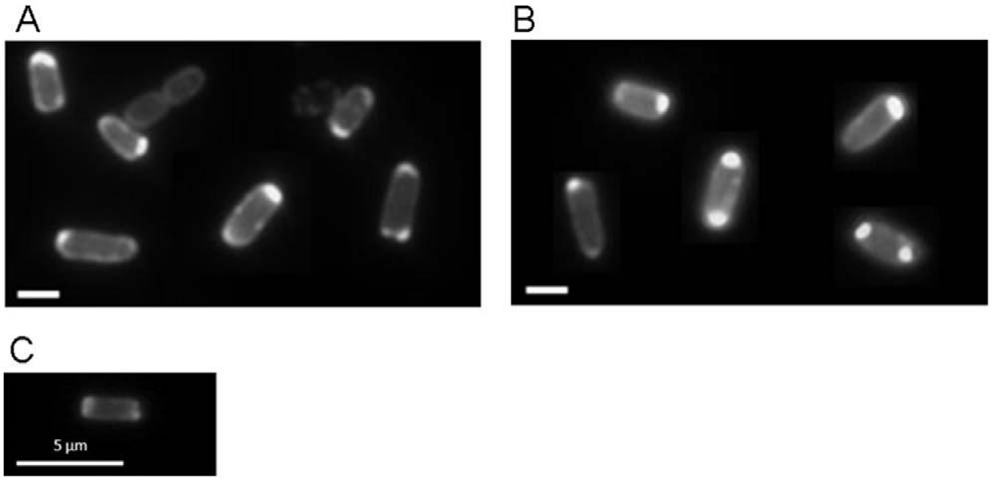
*E. coli* cells expressing DcuS-YFP in *dcuR, dctA* and *dauA* deficient background. DcuS-YFP (pMW407) fluorescence was monitored in *E. coli* IMW238 deficient of *dcuR* (A) or MDO800 deficient of *dctA* (B); scale bars, 1 µm. (C) DcuS-mYFP (pMW1891) fluorescence was monitored in *E. coli* EK1 deficient of *dauA*.

The experiments therefore demonstrate that DcuS is required for polar localization of the other components of the DctA/DcuS/DcuR sensor/regulator complex. Vice versa, deletion of DcuR and DctA had no negative effect on the polar localization of DcuS-YFP (Fig. 4 A, B). The C_4_-dicarboxylate transporter DauA that is active under acidic growth conditions also interacts with the DctA/DcuS sensor unit [41], and it was checked by fluorescence microscopy whether DauA has an effect on the polar localization of DcuS (Fig. 4 C). However, a strain deleted for *dauA* and moderately producing DcuS-mYFP contained the DcuS fusion protein with polar localization very similar to a *dauA* wild-type. Thus, DauA is not required for polar localization of DcuS at neutral pH as well.

### Cellular factors controlling the localization of DcuS-YFP in the bacterial cells

**Figure 5:**
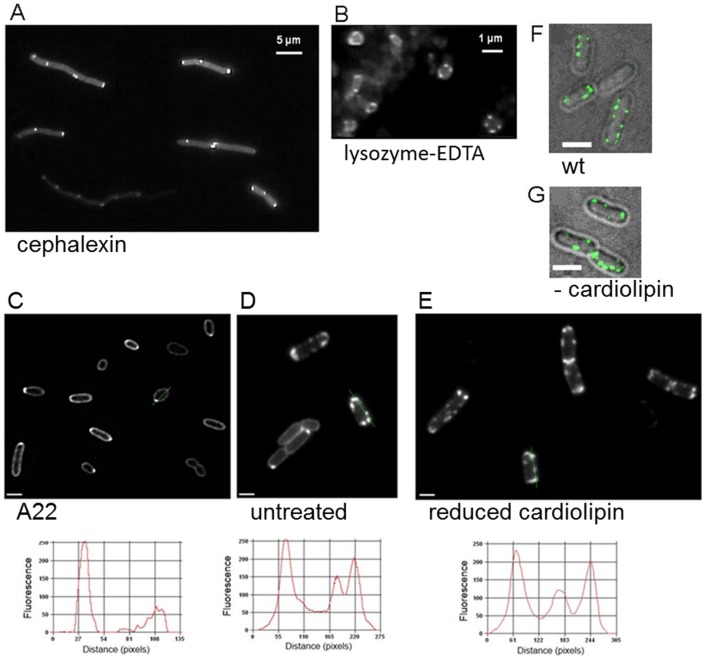
*E. coli* cells expressing DcuS-YFP in filaments, in spheroplasts, and in a cardiolipin-synthase mutant. DcuS-YFP fluorescence (strain IMW262/pMW407) was monitored in *E. coli* cells; (A) treated with cephalexin; (B) treated with Lysozyme-EDTA; (C) treated with A22; (D) without treatment; (E) in a cardiolipin-synthase mutant (strain JW1241/pMW407); (F) DcuS-YFP fluorescence in wild type strain W3110 and (G) in the cardiolipin triple mutant BKT12 Δ*clsABC*; scale bars, 2 µm. For each strain at least 50 cells were monitored with 70 to 95% showing the respective localization. Fluorescence-intensity profiles along the lines indicated in the images C, D, and E are shown in the bottom row.

Various cellular factors including the need for a high degree of cell curvature, specific phospholipids, the bacterial cytoskeleton or the cell division machinery have been discussed or shown to drive specific localization of membrane proteins within the cell [24], [25], [28], [30], [31], [42], [43]. Cellular factors were tested for their effect on the polar accumulation of DcuS that was produced from vector pBAD30. DcuS clusters were still present in *E. coli* filaments that were obtained by treatment of exponentially growing *E. coli* cells with cephalexin (Fig. 5 A). Cephalexin treatment resulted in the formation of long filaments, and the DcuS-YFP clusters were still exclusively located at the poles and the presumed cell division regions where septum formation would take place. Moreover, when spheroplasts, or rounded cells, were formed by treatment of the exponentially growing cells with lysozyme-EDTA, the fluorescence of DcuS-YFP was still arranged in clusters (Fig. 5 B), implying that the arrangement of DcuS in clusters is independent of cell shape. This suggests that intrinsic cellular factors might be responsible for DcuS localization.

It was further investigated if DcuS might be trapped at the cell pole by the anionic phospholipid cardiolipin. Cardiolipin that is found with a mole fraction of 5% from total lipid content in the cytosolic membrane of *E. coli*, is enriched at the cell poles and septa of growing cells [26]. It has been shown that some membranous and cytosolic proteins with polar accumulation [21], [42], [43] require cardiolipin for that location pattern. The location of DcuS-YFP was monitored in a cardiolipin-synthase mutant (Keio strain JW1241) with decreased cardiolipin contents (<0.1% of total phospholipids), and the triple cardiolipin synthase *ΔclsABC* mutant BKT12 that completely lacks cardiolipin [44]. DcuS-YFP clusters in the cardiolipin synthase mutants (Fig. 5 E, G) showed a similar degree of polar accumulation as in wild-type cells or in *dcuS* mutant cells, indicating that cardiolipin is no essential factor for the polar localization of DcuS clusters. For the triple cardiolipin synthase mutant BKT12 a dynamic preferential polar localization was observed similar to that observed for the cardiolipin positive wild-type (Fig. 5 F). This is in contrast to other proteins like the osmosensory transporter ProP or the mechanosensitive channel MscS [42], [43], [45] that requires high cardiolipin contents at the cell poles for polar trapping the proteins. Also, all fusions analysed were as mobile within the cell membrane as in wild type cells (data not shown).

Furthermore, the cytoskeletal filament protein MreB is one of the major and general proteins used for targeted location of proteins in the cell either permanently or during specific growth states. Therefore we tested whether the function of MreB, which itself is helically arranged within the membrane, is required for guiding DcuS-YFP to and localizing it at the cell poles. Since MreB is essential for growth and viability of the cells, the MreB-depolymerizing drug A22 [46] was used to test the significance of functional MreB for DcuS localization. After addition of A22 to exponentially growing cells, the cells became spherical due to MreB depolymerization. DcuS-YFP, however, retained clustering and the clusters were localized to the (presumably previous) cell poles (Fig. 5 C).

### The cytosolic PAS_C_ and kinase domains are required for polar accumulation of DcuS

**Figure 6:**
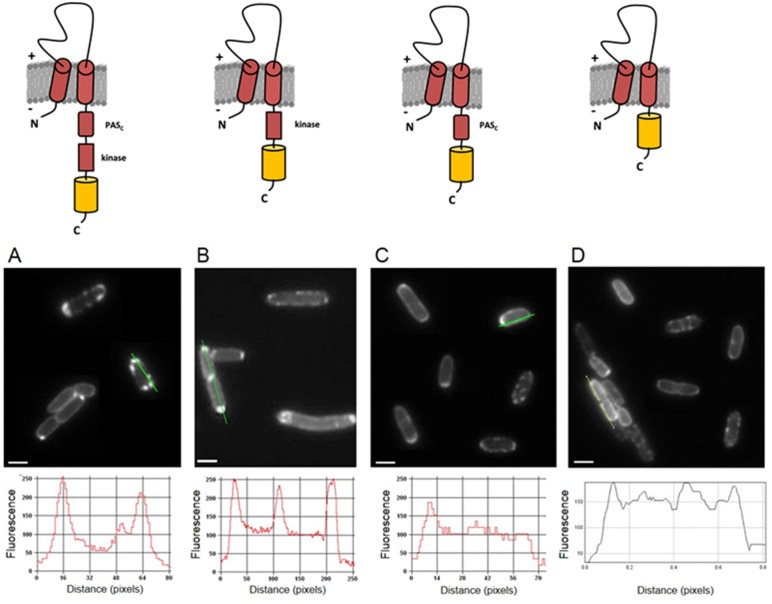
Cellular distribution of truncated variants of DcuS fused to YFP. Upper images indicate the schematic representation of truncated DcuS-YFP variants (DcuS in red; YFP in yellow; membrane in grey; the PAS_C_ and the kinase domain of DcuS are labelled) which were analyzed by fluorescence microscopy as displayed in the respective lower images; (A) full-length DcuS-YFP (strain IMW262/pMW407); (B) DcuS-(ΔPAS_C_)-YFP (strain IMW262/pMW1302); (C) DcuS-PAS_c_-YFP (strain IMW262/pMW1060) (D) DcuS-TM2-YFP (strain IMW262/pMW1061). About 100 cells each were inspected with polar localization of DcuS in approx. 90% of the cells for (A), intermediate localization for 60% in (B), and homogeneous distribution for 80% of the cells in (D). Scale bars, 1 µm. Fluorescence-intensity profiles along the lines indicated in the microscopic images are shown in the bottom row.

DcuS with deleted C-terminal cytoplasmic kinase or kinase plus PAS_C_ domain is still stably inserted in the membrane of *E. coli* [47]. Strains of *E. coli* producing YFP fused to the C-terminal end of full-length DcuS (DcuS-YFP) and to DcuS lacking the kinase or the kinase plus PAS_C_ domains (DcuS-PAS_C_-YFP and DcuS-TM2-YFP, respectively) were examined (Fig. 6). As described before, full-length DcuS-YFP showed clear polar location (Fig. 6 A). A mutant of DcuS with an internal deletion of PAS_C_ showed only a minor loss of the polar localization of DcuS (Fig. 6 B). When the kinase domain was deleted the fusion protein exhibited still preferred polar localization, but the strict clustering was lost and a minor portion of the protein (fluorescence) was smoothly distributed over the cell surface (Fig. 6 C). When the C-terminal deletion of DcuS was extended up to TM2, that is when PAS_C_ and the kinase domains were lacking, most of the fluorescence or of the protein was evenly distributed over the cell membrane with only slight polar preference (Fig. 6 D). The data indicate that presence of the complete cytosolic part of DcuS is required for the polar localization, and that the deletion of individual domains has only a minor effect. Nevertheless, both variants with the C-terminal deletions were still membrane integral and properly folded as suggested by the strong fluorescence [48]. Therefore the cytoplasmic domains of DcuS, i.e. the kinase domain and the PAS_c_ domain, are important for DcuS localization (Fig. 6 A–C). The loss of polar accumulation was independent from the availability of the effector fumarate (not shown). A similar observation on the significance of the cytosolic domains for cellular localization was reported for the chemotaxis receptors, whose cytosolic domains affect their intrinsic cellular distributions [10].

### Co-localization of the related sensor kinases CitA and DcuS in *E. coli*

**Figure 7:**
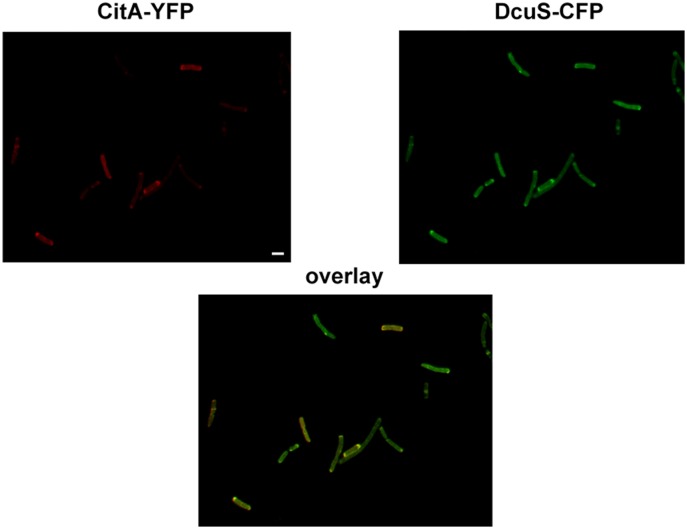
Co-localization of the related sensor kinases CitA and DcuS in *E. coli*. CitA-YFP (pMW442) and DcuS-CFP (pMW408) were coexpressed in IMW262 and fluorescence of YFP (depicted in red) and CFP (depicted in green) were detected separately and merged (overlay image). About 50 cells monitored, with approx. 90% polar localization of the fluorescent proteins. Scale bar, 1 µm.

The citrate-specific sensor histidine kinase CitA, which is closely related to DcuS and belongs to the same family of sensor histidine kinases [21], [49], [50], exhibits polar accumulation within the cytosolic membrane similar to DcuS ([20]; S7 A Fig.). The polar cluster formation is also maintained in filaments (S7 B Fig.), and in A22-treated bacteria (S6 C Fig.) similar to the situation found for DcuS (Fig. 5). Moreover, CitA was shown to interact with DcuS [39]. Consistently, when CitA-YFP and DcuS-CFP were coexpressed, they showed, in addition to polar localization, also co-localization which is in accordance with the direct interaction of both sensor kinases (Fig. 7).

## Discussion

### Polar localization of DctA/DcuS/DcuR Sensor-regulator units

DcuS exhibits dynamic, preferential polar localization even when it is produced at very low levels in a functionally active state. This preferential polar appearance was described earlier in less detailed studies with lower spatial and temporary resolution as a polar localization [20]. The dynamic preferential polar localization observed here is different from the much more static polar localization described for proteins like DivIVa, chemotactic MCPs, or the polar DNA uptake machinery in competent *Bacillus subtilis* cells [51], [52], [53], [54] and might be based on a diffusion and capture mechanism according to Shapiro et al. [55] and Rudner and Losick [56]. MreB is not required for the polar localization, showing that DcuS is also not anchored by this cytoskeletal element.

Part of DctA, presumably the portion that is part of the DctA/DcuS sensor complex [8] and the cognate response regulator DcuR, co-localize with DcuS. Only for DcuS polar localization was an inherent property of the protein whereas DctA and DcuR required presence of DcuS for polar accumulation. DcuS is a minor protein as expected for a sensor, and therefore required overproduction in order to trap the otherwise randomly dispersed (free) DctA-YFP and YFP-linker-DcuR that are not part of the sensor/regulator complex at the poles.

DctA and DcuR obviously reach DcuS by diffusion without guiding by MreB and are then trapped by direct interaction with DcuS. Formation of DctA/DcuS and of DcuS/DcuR complexes has been shown directly by the use of the BACTH and other interaction assays in vivo [8], [40]. The polar clustering observed for DcuS at very low levels and the FRAP experiments confirm that DcuS is able to move rapidly to the cell poles, and that the clustering is not due to molecular crowding, or a concentration artifact. Presence of functional DctA/DcuS/DcuR sensor units is in agreement with the DctA/DcuS and DcuS/DcuR complexes described earlier [8], [9], [40]. Localization of the tripartite sensor complex to the poles relies only on properties of DcuS. The cellular factors responsible for targeting DcuS to the poles are not known as for the chemotaxis sensor complexes and others. Commonly supposed factors for polar accumulation like cardiolipin [42], [43], [45], or cell geometry and high curvature at the cell poles [52], or guiding by the cytoskeleton protein MreB [57] play no direct role for DcuS according to our experimental data. DcuS intrinsic factors for polar localization are the cytoplasmic domains of DcuS, in particular PAS_C_ and the kinase domain. The PAS_C_ domain is also important for the interaction of DcuS with DctA [8]. Phosphorylation of DcuS was no major factor controlling DcuS localization. This is at variance with the developmental regulator DivK (and others) of *C. crescentus* which locates in the phosphorylated state at the cell pole in the stalk region, and in the non-phosphorylated state in the cytoplasm [58], [59]. Therefore, the component or mechanism responsible for the polar localization is not known for DcuS as for many other proteins with polar localization.

### Polar localization of bacterial sensors

For sensors and regulators that control metabolic processes no asymmetric distribution to cell poles or other sites is expected whenever the metabolic processes show no specific localization with the cell apart from cytoplasmic, membraneous or extracytoplasmic localization. For some metabolic pathways including the citric acid cycle an organisation in ‘metabolons’ is discussed which may include specific subcellular localization [2], [60], [61] for optimal spatial organisation of the reactions, or for separation of potentially controversial pathways. Knowledge on the subcellular localization of specific pathways and other cellular processes is only at the beginning and might explain so far unknown localizations of sensory and regulatory pathways, including aerobic and anaerobic C_4_-dicarboxylate metabolism.

The paradigm for sensor proteins with asymmetric localization within *E. coli* and other bacteria is represented by the methyl-accepting chemotaxis proteins (MCPs) [6], [62]. MCP clusters consist of a large number of receptors and are predominantly observed near the cell poles. The organisation of the receptors in clusters supports stimulus integration and increased sensitivity of the sensors [11], [63]. The localization pattern of DcuS resembles that of the well-characterized chemotaxis sensors in many aspects. Both sensor types have in common perceiving periplasmic substrates as signals. The DcuS and the MCP sensory systems and complexes, however, are independent from each other, and polar clustering of DcuS is retained in a ΔMCP strain lacking the methyl-accepting chemotaxis proteins [20]. Overall, it turns out that metabolic sensor systems, like DcuS/DcuR, or others, like the MCP complexes, have polar localization for reasons that have not been identified so far.

## Supporting Information

S1 Fig Functional test of DcuS-mVenus fusion protein by induction of dcuB’–‘lacZ expression. The *E. coli* Strains were grown anaerobically in eM9 medium supplemented with glycerol (50 mM) and dimethyl sulfoxide (20 mM) with (dark grey bars) and without disodium fumarate (20 mM) to the exponential growth phase. β-Galactosidase activity assays were conducted as described by Monzel et al. [S2]. Wt: IMW237; DcuR-: IMW238; DcuS-mVenus: IMW612; DcuR−/p(DcuR): IMW238 pMW1740; DcuS-mVenus/p(DcuR): IMW612 pMW1740.
(TIF)

S2 Fig Localization of chromosomally expressed DcuS-mVenus in *E. coli*. DcuS-mVenus was chromosomally expressed from the native *dcuS* promoter (strain IMW612/pDS132::*dcuS-mvenus*) in exponentially growing cells and fluorescence was visualized; left panel, deconvoluted fluorescence channel; right panel, overlay of fluorescence and DIC image of representative cells. Scale bars, 2 µm.
(TIF)

S3 Fig Localization of DcuS-YFP in *E. coli*. DcuS-YFP fluorescence (strain IMW262/pMW407) was visualized; (A) overview image of induced cells (conditions as described in Materials and Methods); (B) uninduced cells in early-exponential growth phase. Scale bars, 2 µm.
(TIF)

S4 Fig Aerobic growth on fumarate by strains containing YFP/DcuR fusion proteins. Cells were grown aerobically in eM9 medium containing disodium fumarate (50 mM). Only the strain containing the mYFP(A206K)-linker-DcuR fusion (pMW1953) was able to complement the DcuR deficiency of IMW238. It shows similar growth as wildtypic DcuR (pMW1740). Strains expressing DcuR-YFP (pMW1739) or YFP-DcuR (pMW1741) displayed deficient growth. The *dcuR* gene was amplified by PCR from plasmid pMW180 with oligonucleotide primers dcuRpetCfor (5′-GCGGCAGCCATATGATCAATG-3′) and dcuRSacIrev (5′-GCAATAGAGCTCCAGTAGTGAG-3′). The PCR fragment was cloned into pMW391 via NdeI and SacI, resulting in pMW1081. *dcuR-yfp* was finally cloned from pMW1081 via XbaI into pMW643, resulting in pMW1739. The construct encodes His_6_-DcuR(1-234)-(EL)-YFP(4-240) referred to as DcuR-YFP. Additionally *dcuR* was cloned into plasmid pMW643 via XbaI resulting in pMW1740.
(TIF)

S5 Fig Functional test of YFP/DcuR fusion proteins by induction of *dcuB’–‘lacZ* expression. *E. coli* strain IMW238 [MC4100 *dcuR*::Kan^r^, λ(Φ*dcuB’-‘lacZ*)] was grown anaerobically in eM9 medium containing glycerol (50 mM) and dimethyl sulfoxide (20 mM) as growth substrates with (dark grey) and without fumarate (light grey) (20 mM) as effector. DcuR-: IMW238; DcuR: IMW238 pMW1740; DcuR-YFP: IMW238pMW1739; YFP-DcuR: IMW238 pMW1741, mYFP(A206K)-linker-DcuR fusion: IMW238 pMW1953.
(TIF)

S6 Fig Coexpression of YFP and DcuS-CFP. YFP (pMW765) and DcuS-CFP (pMW408) were coexpressed in IMW262 and fluorescence of YFP (depicted in red) and CFP (depicted in green) were detected separately and merged (overlay image). White arrows indicate polar regions where YFP is excluded by DcuS-CFP. Scale bar, 1 µm.
(TIF)

S7 Fig Polar localization of the related sensor kinase CitA fused to YFP. CitA-YFP fluorescence (strain IMW279/pMW442) was visualized; (A) in untreated cells; (B) in cells treated with cephalexin; (C) in cells treated with A22. Scale bars, 1 µm.
(TIF)

S1 Table Functional test for DcuS-Bs2 *in vivo* by reporter gene measurement of *dcuB-lacZ*. *E. coli* IMW260 containing the plasmids shown in the table or IMW237 was grown anaerobically in eM9 medium [S1] containing glycerol (50 mM) and dimethyl sulfoxide (20 mM) as growth substrates with and without fumarate (20 mM) as effector. Activities (in Miller Units, MU) are shown as the average of at least four independent experiments. The standard deviation is shown.
(DOCX)

S1 Movie Time lapse experiments DcuS-YFP. Time lapse experiments of exponentially growing *E. coli* JM109pMW407 expressing DcuS-YFP, time intervals 2 s, shown are 5 frames/s.
(AVI)

S2 Movie Time lapse experiments CitA-YFP. Time lapse experiments of exponentially growing *E. coli* expressing CitA-YFP (plasmid 442), exposures every 2 seconds, 5 frames/s.
(AVI)

S1 Text Construction of the chromosomal *dcuS-mVenus* fusion.
(DOCX)
