# Supplementary figures and images for: Polar Localization of a Tripartite Complex of the Two-Component System DcuS/DcuR and the Transporter DctA in Escherichia coli Depends on the Sensor Kinase DcuS
Source: PLoS One. 2014 Dec 30;9(12):e115534. doi: 10.1371/journal.pone.0115534 (PMC4280142; doi:10.1371/journal.pone.0115534)

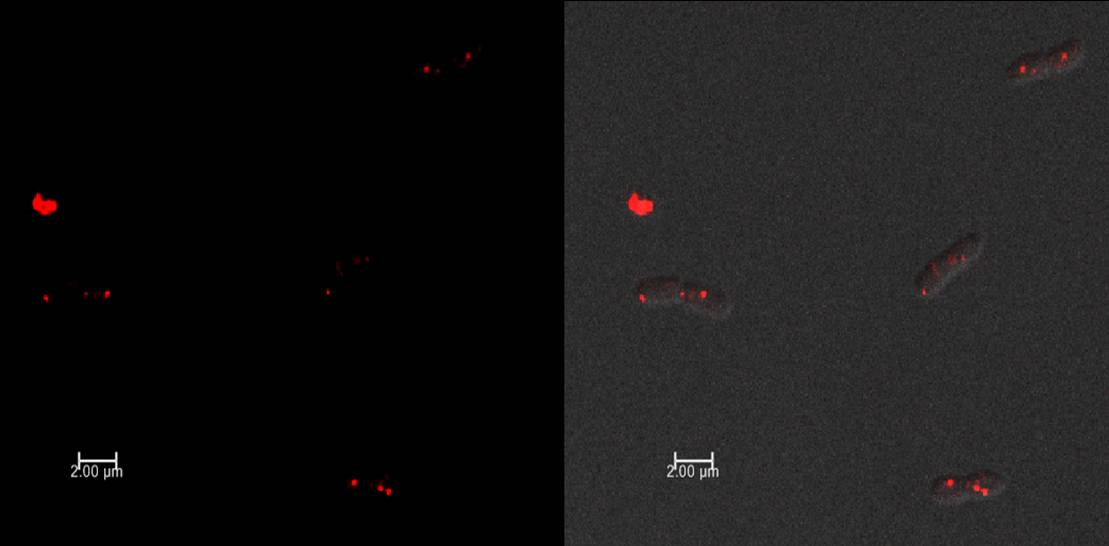

Supplement: S2 Fig — Localization of chromosomally expressed DcuS-mVenus in E. coli. DcuS-mVenus was chromosomally expressed from the native dcuS promoter (strain IMW612/pDS132::dcuS-mvenus) in exponentially growing cells and fluorescence was visualized; left panel, deconvoluted fluorescence channel; right panel, overlay of fluorescence and DIC image of representative cells. Scale bars, 2 µm. (TIF) [file pone.0115534.s002.tif]

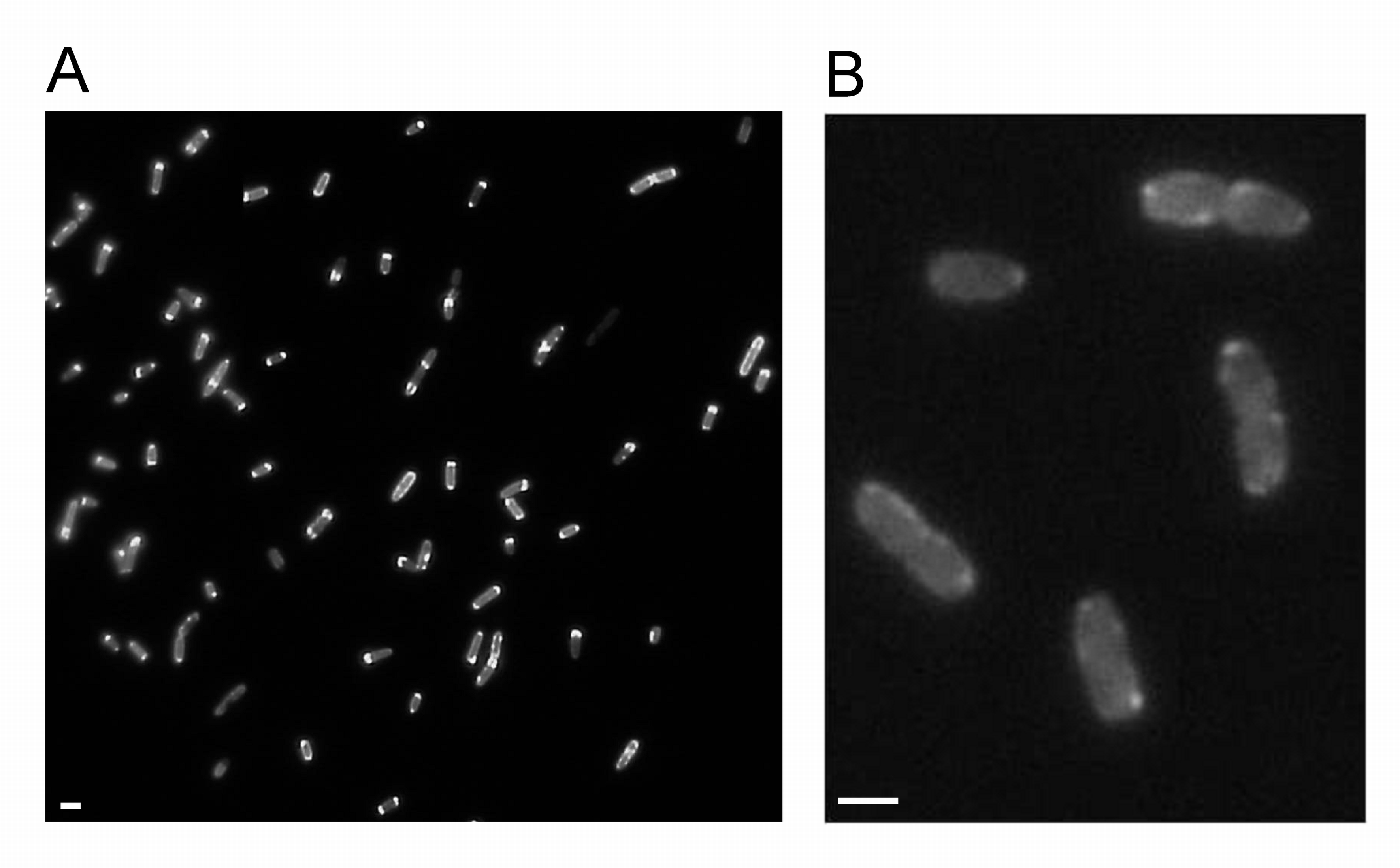

Supplement: S3 Fig — Localization of DcuS-YFP in E. coli. DcuS-YFP fluorescence (strain IMW262/pMW407) was visualized; (A) overview image of induced cells (conditions as described in Materials and Methods); (B) uninduced cells in early-exponential growth phase. Scale bars, 2 µm. (TIF) [file pone.0115534.s003.tif]

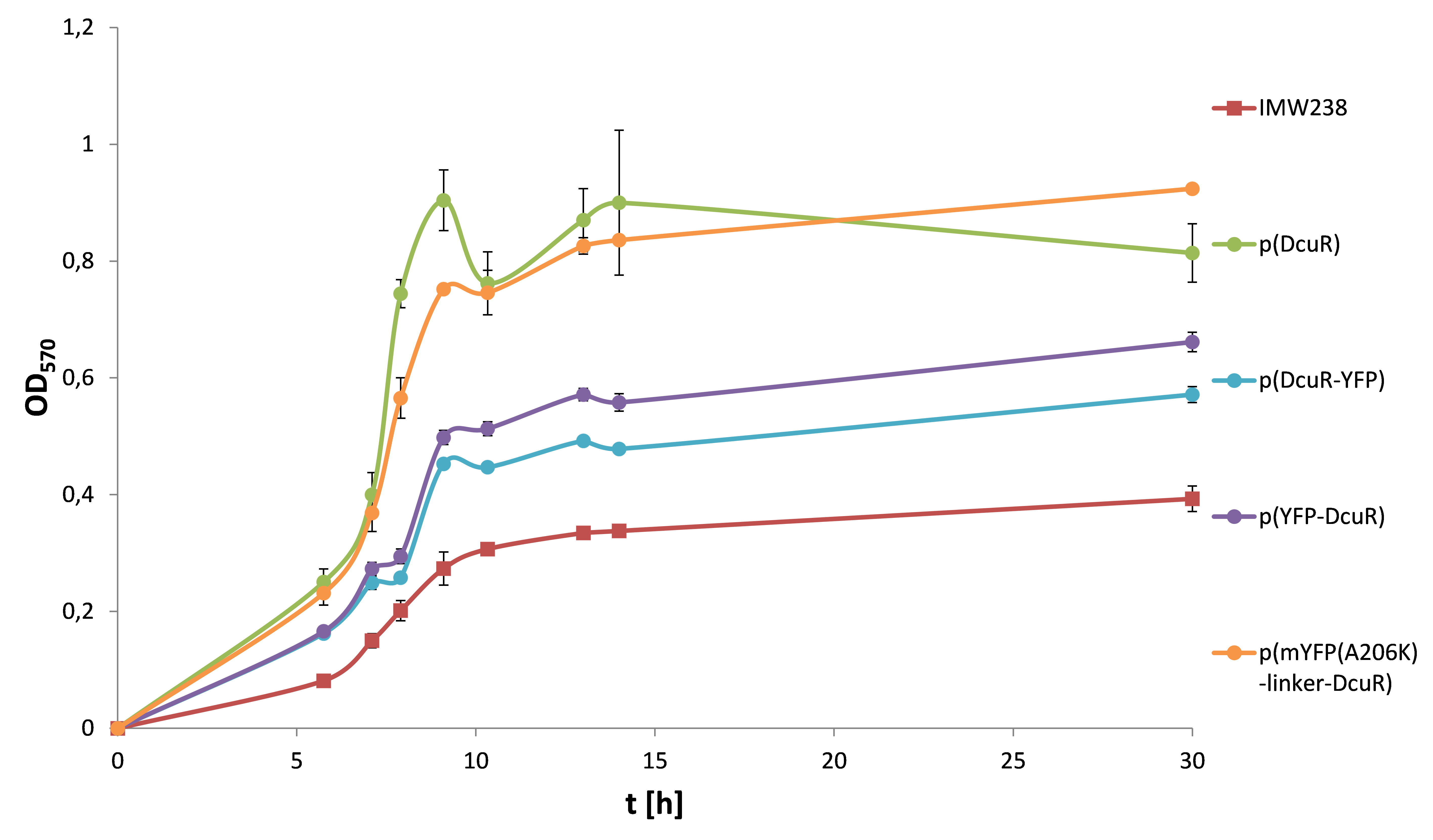

Supplement: S4 Fig — Aerobic growth on fumarate by strains containing YFP/DcuR fusion proteins. Cells were grown aerobically in eM9 medium containing disodium fumarate (50 mM). Only the strain containing the mYFP(A206K)-linker-DcuR fusion (pMW1953) was able to complement the DcuR deficiency of IMW238. It shows similar growth as wildtypic DcuR (pMW1740). Strains expressing DcuR-YFP (pMW1739) or YFP-DcuR (pMW1741) displayed deficient growth. The dcuR gene was amplified by PCR from plasmid pMW180 with oligonucleotide primers dcuRpetCfor (5′-GCGGCAGCCATATGATCAATG-3′) and dcuRSacIrev (5′-GCAATAGAGCTCCAGTAGTGAG-3′). The PCR fragment was cloned into pMW391 via NdeI and SacI, resulting in pMW1081. dcuR-yfp was finally cloned from pMW1081 via XbaI into pMW643, resulting in pMW1739. The construct encodes His6-DcuR(1-234)-(EL)-YFP(4-240) referred to as DcuR-YFP. Additionally dcuR was cloned into plasmid pMW643 via XbaI resulting in pMW1740. (TIF) [file pone.0115534.s004.tif]

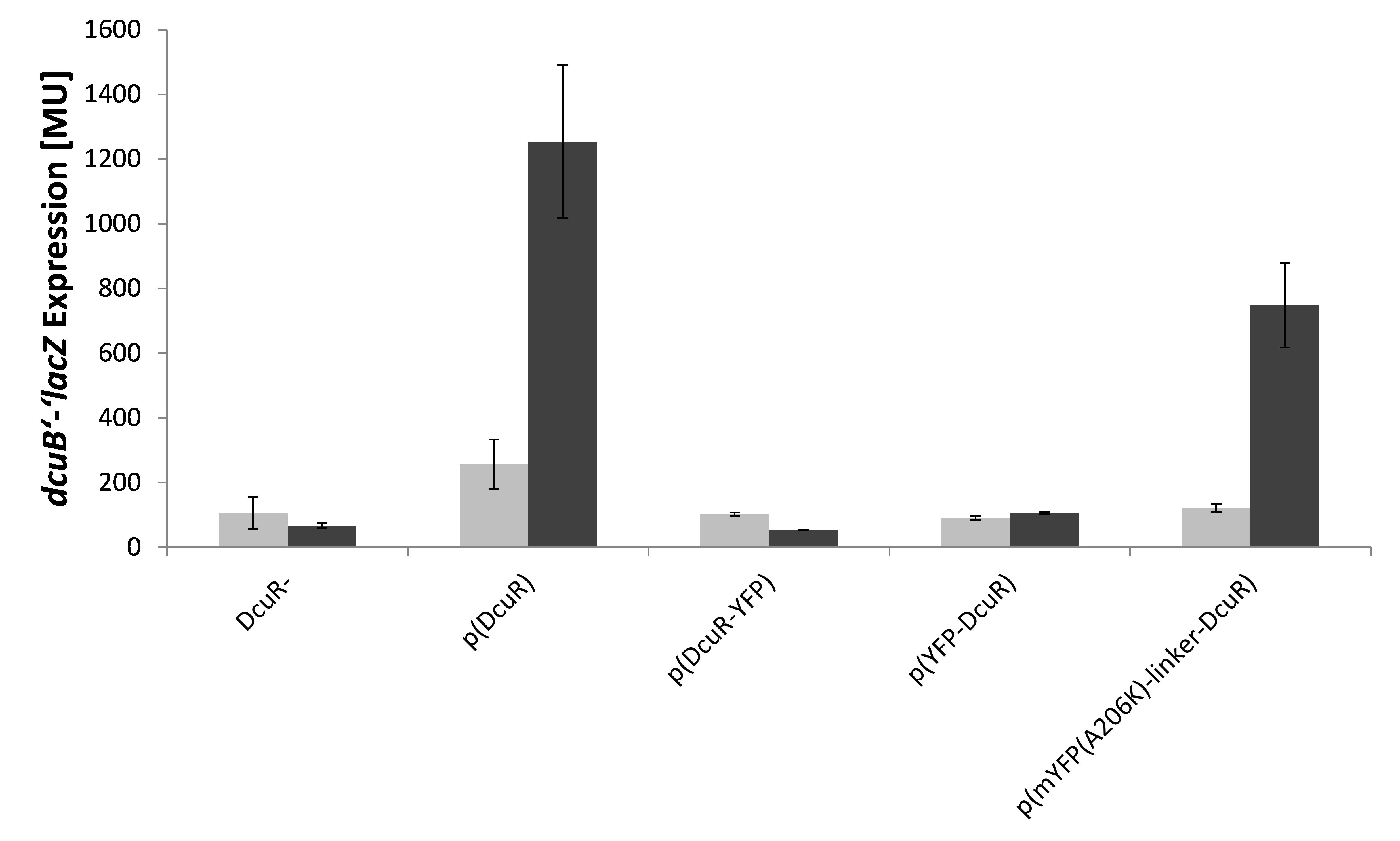

Supplement: S5 Fig — Functional test of YFP/DcuR fusion proteins by induction of dcuB’–‘lacZ expression. E. coli strain IMW238 [MC4100 dcuR::Kanr, λ(ΦdcuB’-‘lacZ)] was grown anaerobically in eM9 medium containing glycerol (50 mM) and dimethyl sulfoxide (20 mM) as growth substrates with (dark grey) and without fumarate (light grey) (20 mM) as effector. DcuR-: IMW238; DcuR: IMW238 pMW1740; DcuR-YFP: IMW238pMW1739; YFP-DcuR: IMW238 pMW1741, mYFP(A206K)-linker-DcuR fusion: IMW238 pMW1953. (TIF) [file pone.0115534.s005.tif]

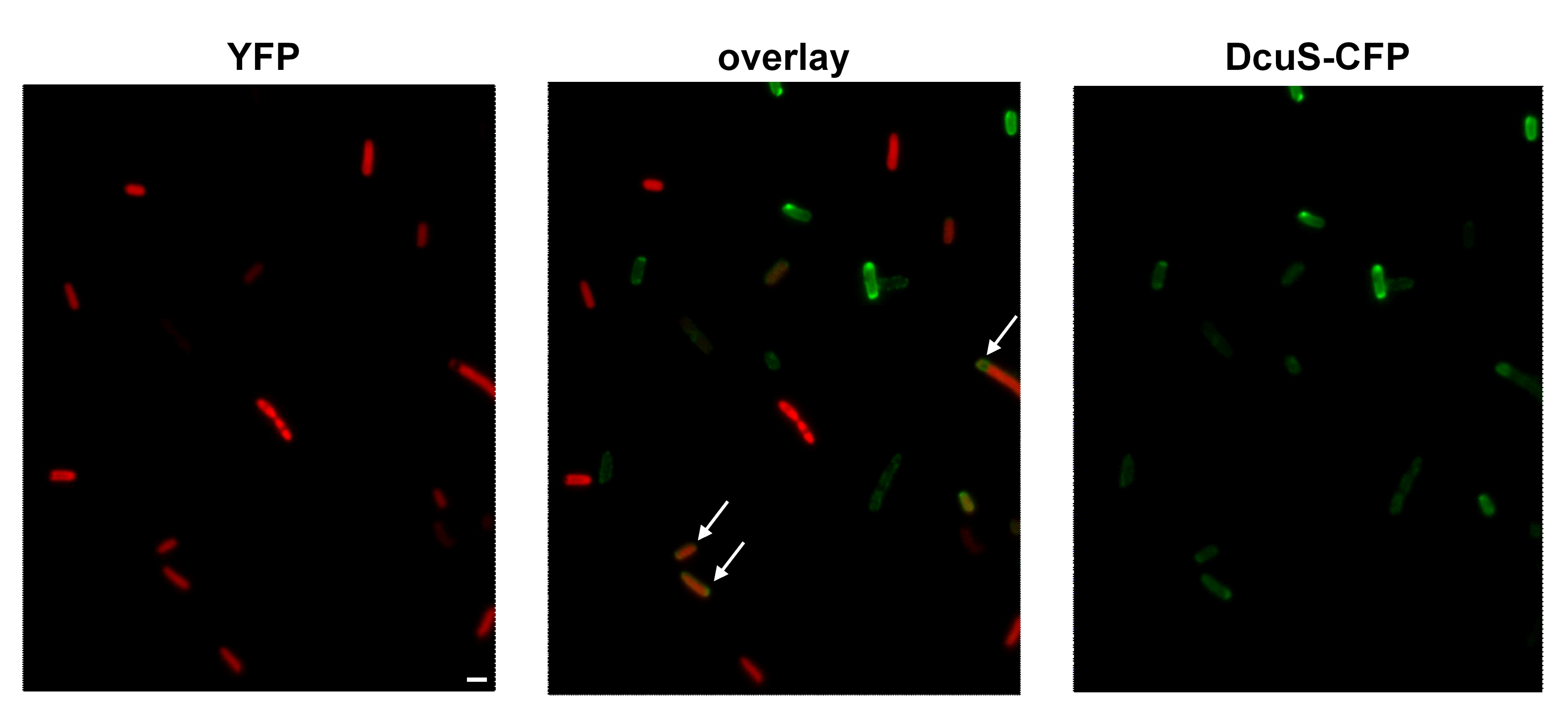

Supplement: S6 Fig — Coexpression of YFP and DcuS-CFP. YFP (pMW765) and DcuS-CFP (pMW408) were coexpressed in IMW262 and fluorescence of YFP (depicted in red) and CFP (depicted in green) were detected separately and merged (overlay image). White arrows indicate polar regions where YFP is excluded by DcuS-CFP. Scale bar, 1 µm. (TIF) [file pone.0115534.s006.tif]

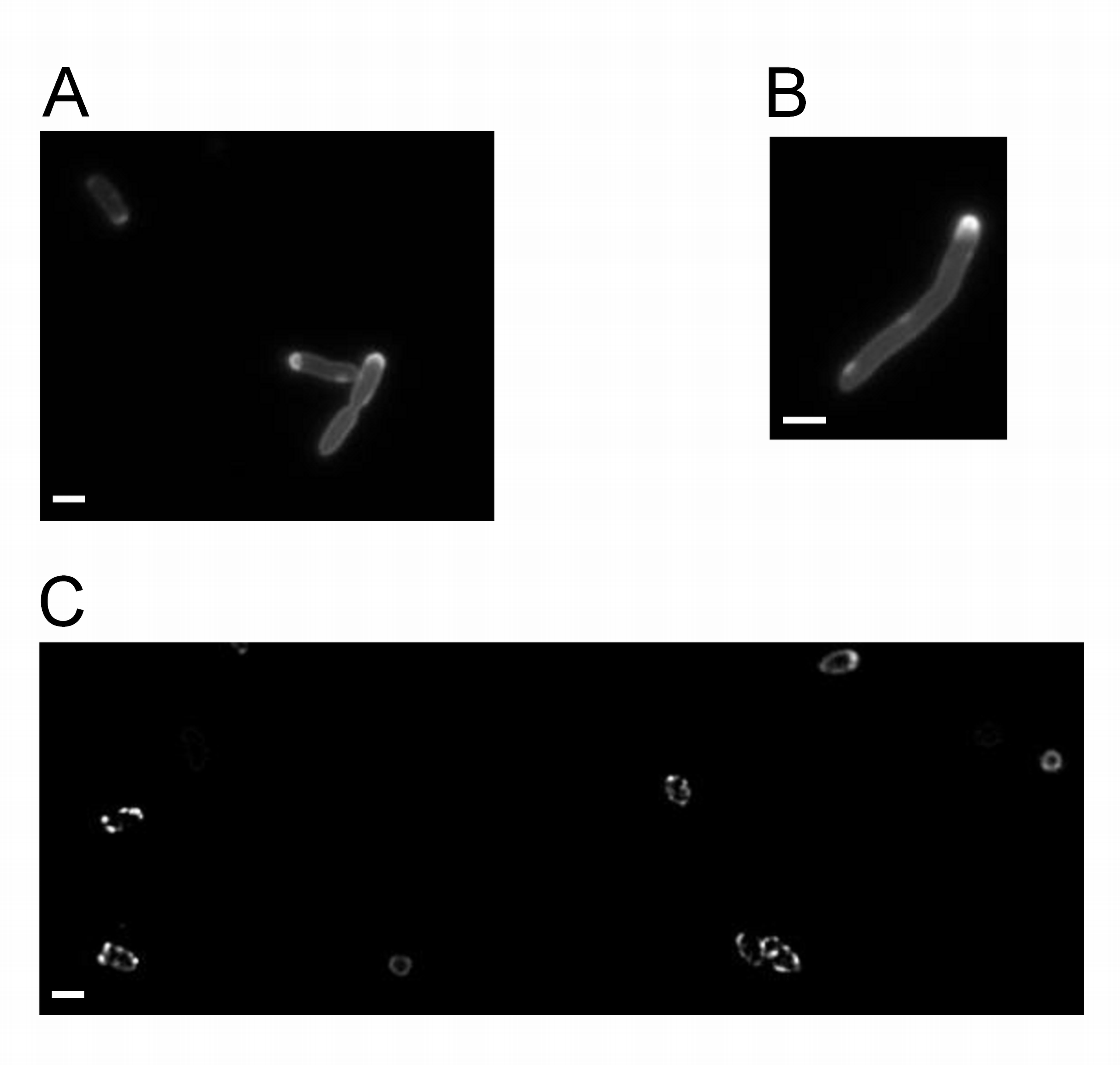

Supplement: S7 Fig — Polar localization of the related sensor kinase CitA fused to YFP. CitA-YFP fluorescence (strain IMW279/pMW442) was visualized; (A) in untreated cells; (B) in cells treated with cephalexin; (C) in cells treated with A22. Scale bars, 1 µm. (TIF) [file pone.0115534.s007.tif]
