# Supplementary material for: Polar Localization of a Tripartite Complex of the Two-Component System DcuS/DcuR and the Transporter DctA in Escherichia coli Depends on the Sensor Kinase DcuS
Source: PLoS One. 2014 Dec 30;9(12):e115534. doi: 10.1371/journal.pone.0115534 (PMC4280142; doi:10.1371/journal.pone.0115534)
Supplement: S1 Table — Functional test for DcuS-Bs2 in vivo by reporter gene measurement of dcuB-lacZ. E. coli IMW260 containing the plasmids shown in the table or IMW237 was grown anaerobically in eM9 medium [S1] containing glycerol (50 mM) and dimethyl sulfoxide (20 mM) as growth substrates with and without fumarate (20 mM) as effector. Activities (in Miller Units, MU) are shown as the average of at least four independent experiments. The standard deviation is shown. (DOCX) [file pone.0115534.s008.docx]

| Effectors | *dcuB-lacZ* (MU) | | | |
| --- | --- | --- | --- | --- |
|  | IMW260  (Δ*dcuS*) | IMW260pMW181  (Δ*dcuS* p*dcuS*) | IMW260pMW875  (Δ*dcuS* p*dcuS-bs2*) | IMW237  (Wild-type) |
| H_2_O | 26 ± 6 | 17 ± 4 | 23 ± 5 | 35 ± 6 |
| Fumarate | 23 ± 11 | 464 ± 17 | 1065 ± 130 | 1040 ± 125 |
