# Supplementary material for: Polar Localization of a Tripartite Complex of the Two-Component System DcuS/DcuR and the Transporter DctA in Escherichia coli Depends on the Sensor Kinase DcuS
Source: PLoS One. 2014 Dec 30;9(12):e115534. doi: 10.1371/journal.pone.0115534 (PMC4280142; doi:10.1371/journal.pone.0115534)
Supplement: S1 Text — Construction of the chromosomal dcuS-mVenus fusion. (DOCX) [file pone.0115534.s011.docx]

**Construction of the chromosomal *dcuS-mVenus* fusion.** The chromosomal *dcuS-mVenus* fusion was constructed by replacing *bs2* with *mvenus* in a *dcuS-bs2* fusion. The *dcuS* gene was amplified from genomic DNA of *E. coli* MG1655 by PCR with oligonucleotide primers dcuSsurHind3-for (5’-CCTATCAAGCTTAAACGCTCTGG-3’) and dcuSsurHind3-rev (5’-GAAGGATGAAGCTTGTGCCAGG-3’). The PCR fragment was cloned via *Hind*III into pUC18. *bs2* was amplified from pGlow-Bs2-stop with primers Bs2-stop-for (5’-CGAGCTCCATATGGCGTCG-3’) and Bs2-stop-rev (5’-GCATGCCTGCAGTCACTCG-3’) and cloned in fusion to *dcuS* via restriction sites *Nde*I and *Pst*I, resulting in pMW842. Prior to the cloning of *bs2*, *Nde*I and *Pst*I restriction sites were mutated from pUC18 with primer Mut_puC_NdeI-for (5’-GTATTTCACACCGCACATGGTGCACTCTCAG-3’) and complementary primer Mut_puC_NdeI-rev, and Mut_puC_PstI-for (5’-CTAGAGTCGACCCGCAGGCATGCAAGC-3’) and complementary primer Mut_puC_PstI-rev, respectively, by site-directed mutagenesis (QuikChange kit, Stratagene). A *Nde*I restriction site was then inserted at the 3’-end of *dcuS* by simultaneous mutation of the *dcuS* stop codon with mutagenesis primer Mut_sur_NdeI-for (5’-GGAGAGGTCGAACAGAGGACATATGTATTAATTATCGATGACGACGC-3’) and complementary primer Mut_sur_NdeI-rev. 13 bp upstream of the inserted *Nde*I site, a *Pst*I restriction site was inserted with mutagenesis primer Mut_PstI-for (5’-GTCGAACAGAGGACATATGTATTAATTATCGCTGCAGACGCAATGG-3’) and complementary primer Mut_PstI-rev. In this way, *bs2* could be cloned in fusion to *dcuS*.

The *bs2* gene and adjacent sequences of *dcuS* upstream and downstream of *bs2*, was amplified from pMW842 with primers dcuS-Bs2SacI-for (5’-CTACGAGAGCTCCACGCTG-3’) and dcuS-Bs2SacI-rev (5’-CAGTGCCGAGCTCGTGCC-3’) and inserted into pDS132 via *Sac*I, resulting in pMW843. The *mvenus* gene was amplified from pSG1164::mVenus with primers mVenus-for (5’-CCGCATCATATGGTGAGCAAGGGCGAGG-3’) and mVenus-SDr-rev (5’-CCCTATCTCGAGC*CAT*CTGTTCGACCTCTCCCCGTCTTACTTGTACAGCTCGTCCATGC-3’). Subsequently, the PCR fragment was cloned via *Nde*I and *Xho*I into pMW843 and simultaneously *bs2* was cut out of pMW843 by *Nde*I and *Xho*I, finally resulting in pDS132::*dcuS-mvenus*. Due to the conditional R6K origin of replication of pDS132, which requires the π protein (encoded by the *pir* gene) for replication, cloning into pDS132 was performed in *E. coli* JM109λ*pir*. The suicide vector was used to insert *dcuS-mvenus* into the chromosome of IMW237 via homologous recombination, resulting in IMW612. Besides, *dcuS-bs2-stop* was cloned into the low-copy vector pMW643 under control of the *dcuS* promoter. The gene fusion was amplified from pMW842 with primers dcuS-bs2_Eco-for (5’-CACAAAGAGAATTCCAGCG-3’) and dcuS-bs2_Eco-rev (5’-CCTGGGAATTCTGCTACG-3’) and cloned into pMW643 using restriction endonuclease *Eco*RI, resulting in pMW875. pMW875 carries a tetracycline resistance gene that allows the selection of the plasmid for studies of the functional state of *dcuS* within the gene fusion. The *dcuS-bs2* fusion encodes DcuS(1-543)-(GH)-Bs2(1-137).
Homologous recombination into the chromosome of *E. coli* strains: Homologous recombination into the chromosome of *E. coli* was achieved using the suicide vector pDS132 (57). *E. coli* MC4100 or IMW237, both lacking the *pir* gene, was transformed with pMW843 or pDS132::*dcuS-mvenus*, respectively. Transformed cells were plated on LB-chloramphenicol plates to select plasmid integration into the recipient chromosome. After overnight growth at 37 °C, one colony was picked, diluted in 10 mM MgSO_4_, and serial dilutions were plated on LB agar plates containing 5 % sucrose and without NaCl. This plating step allowed selection of plasmid excision from the chromosome by a second crossover event, leading to allelic exchange. After overnight incubation at 37 °C, three colonies were streaked on chloramphenicol-containing LB agar plates and in parallel on LB agar plates with 5 % sucrose and without NaCl. Sucrose-resistant and chloramphenicol-sensitive colonies were stored in a glycerol suspension at -80 °C. Since allelic exchange depends on the location of the second crossover event, picked colonies were screened by PCR in order to identify those carrying the desired allele (statistically 50 % of the colonies), resulting in IMW570 or IMW612, respectively. Expression of the *dcuB*-*lacZ* reporter gene fusion was determined by measuring the β-galactosidase activity (Table S1) of exponentially growing cultures (OD_578_ of 0.5-0.8) at 37 °C under anaerobic conditions in enriched mineral (eM9) medium supplemented with acid-hydrolyzed casamino acids (0.1%) and tryptophan (0.005%); glycerol (50 mM) and dimethyl sulfoxide (50 mM) with or without sodium fumarate (20 mM) were used as the substrates. For anaerobic growth, cultures were incubated in degassed medium in rubber-stoppered infusion bottles under N_2_. β-Galactosidase activities were measured at least in triplicate for each experiment (S1, S2).

**References:**

S1 Miller JH **(**1992) A short course in bacterial genetics. Cold Spring Harbor Laboratory Press, Cold Spring Harbor, NY

S2 Monzel C, Degreif-Dünnwald P, Gröpper C, Griesinger C, Unden G (2013) The cytoplasmic PAS_C_ domain of the sensor kinase DcuS of *Escherichia coli*: role in signal transduction, dimer formation, and DctA interaction. Microbiologyopen 2: 912-927.
